# Positive and psycho-pathological aspects between shame and shamelessness

**DOI:** 10.3389/fpsyg.2022.941576

**Published:** 2022-08-03

**Authors:** Anna Saya, Gregorio Di Ciaccia, Cinzia Niolu, Alberto Siracusano, Marianna Melis

**Affiliations:** ^1^Psychiatry Unit, Department of Systems Medicine, University of Rome Tor Vergata, Rome, Italy; ^2^Psychiatry and Clinical Psychology Unit, Department of Neurosciences, Fondazione Policlinico Tor Vergata, Rome, Italy; ^3^Istituto Freudiano per la Clinica, la Terapia e la Scienza, Rome, Italy; ^4^Azienda Sanitaria Universitaria Friuli Centrale, Udine, Italy

**Keywords:** shame, relationships, belongingness, emotion acceptance, psychosis, eating disorders, personality disorders, depression

## Abstract

Interpersonal relationships represent an essential aspect of mental wellbeing and social functioning. If all the symptoms contain a relational meaning, shame represents the relational affect par excellence both in terms of its origin and its purpose. This paper aims to highlight the role of shame as an affect inherent in the rhythmic nature of the encounter with the other, as well as the pathological elements of this aspect in both its conscious and unconscious dimensions. There is a heterogeneous quantitative and qualitative declination of shame, or of the defenses against this affect, among the various pathologies. We consider the fundamental needs of belonging and acceptance and the parallel abandonment anguish from various psychoanalytic and philosophical theoretical perspectives and then analyze the link between their dissatisfaction and the origin of shame. We also touch on the different interpretaions of shame based on eastern and western cultural norms. These hypotheses are closely intertwined with the beliefs of classical psychopathology. The role of the body in the encounter with the other and in the experience of shame is also examined. In particular, we study the role of this affect in schizophrenia, depression, eating disorders, and personality disorders.

## Introduction

In the book of Genesis, shame and the consequent need to hide one's body parts seems to represent the relational clock of the possibility of contact when the previously idealized symbiosis was interrupted. In a similar light, this paper aims to highlight the role of shame as an affect inherent in the rhythmic nature of the encounter with the other, as well as the pathological element of this aspect in both its conscious and unconscious dimensions. From a review of the psycho-dynamic, philosophical, and psycho-pathological literature, it emerges that the emotion of shame can play either a positive or a psycho-pathological role. The latter is more commonly associated with the western world while eastern cultures tend to regard it as exclusively positive. We highlight the hypotheses inherent to shamelessness (i.e., a deficit of shame), a common characteristic of psychosis and psychopathy, as the corollary of the peculiarity of the object relations of psychotic subjects.

In the psycho-dynamic field, Wurmser notes that shame shows a continuum between two poles that represent two extreme modes of functioning (Levin and Wurmser, 1996). On one end is the constitutive modulation of interpersonal relationships. At the other end is a feeling of shame crystallized over time, which contains a consequent structure of defenses that configure a “mental disorder”, whether psychotic or neurotic, with different mechanisms and heterogeneous roles of shame. Moreover, its symptomatology can be permeated by an excessive tendency to hide, or by its counterpoint, “shamelessness”, both consequences of shame (Levin and Wurmser, 1996).

To explain this continuum more specifically: on one end there is the anguish signal of a state of tension guiding the Ego to reducing arousal by some suitable means such as a conscious renunciation or avoidance of situations of exposure (Levin and Wurmser, 1996). At the extreme end of this continuum is the traumatic pole that has an instinctual quality and can produce a paralyzing inhibition that can serve partly for the purpose of discharge but at the same time to isolate the person from danger (Levin and Wurmser, 1996). Therefore, if both signal and archaic anguish (i.e., the anguish of shame) tend toward a protective function, the latter determines a reaction of concealment and blocking of perspective activities generally used for drive purposes (or a counter-phobic attitude of pathological lack of shame) while the former would pursue the realization of relational desire. A corollary of these two poles could be the reality principle and the pleasure principle with its consequent defensive barrier. The latter can take place in a form of concealment that Wurmser identifies as a “proto-defense”, a very archaic form of restriction against the more global drive demands of fusion with the object (Levin and Wurmser, 1996).

From a psycho-dynamic point of view, all symptoms contain a relational meaning. Among these, shame seems to represent relationality more explicitly. That is to say, the presence of the other, real or imaginary, is an essential prerequisite for this affect. Shame is the relational affect par excellence. It is defined as a “disturbance or sense of unworthiness of the subject who presumes to receive or actually receives disapproval of his state or conduct from others” (Galimberti, 1992). “The Self is exposed to itself, that is, we are capable of viewing ourselves. A self capable of self-reflection is unique to humans” (Lewis, 1992). Compare by contrast the following remark by Darwin, cited by Zahavi: “[i]t is not the simple act of reflecting on our own appearance, but the thinking what others think of us, which excites a blush” (Zahavi, 2014). The presence of the other in the analysis of shame seems to be the common denominator between heterogeneous theoretical contexts.

The theme of shame is treated extensively in the philosophical field, particularly by Sartre, Scheler, and Zahavi. Zahavi notes that Sartre also believes that shame is an emotion that reveals our relationality, stating that “[f]or Sartre, the body symbolizes our defenseless state as objects. To put on clothes is to attempt to hide one's object-state; it is to claim the right of seeing without being seen, that is, to be a pure subject” (Zahavi, 2014). He goes on to note that “Sartre argues that the feeling of shame refers to the other-as-subject and that the other-as-subject can be present even when the other-as-object is absent and he or she has internalized the perspective of the others” (Zahavi, 2014). Zahavi (2014) asserts that according to Sartre, shame arises from the human condition of being an object who accepts the evaluating gaze of the other and that this acceptance of another's viewpoint creates recognition of oneself as no longer being the “temporal and spatial center of the world”. In the philosophical field, the role of the body in the relationality of this affect is specifically emphasized.

Recent research that applies the evolutionary perspective to emotions, hypothesizes the central role of shame in the regulation of behaviors delegated to the identity and maintenance of the group, to the social bond, and competition for mating (Gilbert and Andrews, 1998). Regarding the anguish signal from an evolutionary point of view, most negative affects should have some capacity to turn off, interrupt, or dampen the positive affect because negative affects usually act to alert animals to dangers and engage in defensive behaviors. Although this emotion contains an unpleasantly dysphoric state, it is precisely this anguish signal that triggers actions aimed at avoiding exclusion from the group, so the individual maintains its protection. This mechanism appears in some way universal and, in fact, responds to species-specific conservative motivations. Shame plays a useful, even essential, role in guiding an individual's behavior to match well with the values of their particular group (Gilbert and Andrews, 1998). Zahavi (2014) notes that Scheler, Strauss, and Bollnow underline the positive influence of shame which plays a protective role in the most intimate and private aspects. This element would be an essential prerequisite for good social functioning. In particular, Scheler (1987) connects shame to the emergence of conscience and ethics “[t]he shame reaction must be seen in the light of a normative commitment that existed prior to the situation about which one is ashamed” and argues that the occurrence of shame testifies to the presence of a certain kind of self-respect and self-esteem.

The intertwining of large areas of overlap between classical psychopathology and psychoanalysis concerning the fundamental role of shame in some mental illnesses is greatly interesting. We can ask ourselves about the role of shame in disorders characterized by a peculiar declination of separation anxiety such as psychosis (etiopathogenetic theory on the deficient primary symbiotic phase in schizophrenia), eating disorders (especially bulimia nervosa), disorders macroscopically related to trauma (for instance, physical or sexual child abuse), personality disorders (in particular, borderline personality disorders), severe depression (especially in adolescence), and trichotillomania. In the previous examples, some authors postulate that childhood sexual abuse is the link between shame and disorders (Gilbert and Andrews, 1998).

However, the role of sexual abuse in dysmorphophobia, paranoia, and social phobia is debated. Some authors consider the role of sexual abuse to be insignificant while others tend to emphasize it. One study which highlights how emotional abuse, even more so than neglect, asserts that such abuse is an anamnestic predictor of social anxiety symptoms as it generates shame-proneness which in turn determines self-criticism (which can be considered a strategic defense toward the experience of shame) which underlies the symptoms of social anxiety (Shahar et al., 2015). “Specifically, it is suggested that early experiences of maltreatment contribute to the development of SAD *via* the internalization of a shame-based cognitive affective schema, characterized by an overall sense of inadequacy” (Shahar et al., 2015). In line with previous findings, this study highlighted the significant correlation between emotional abuse and symptoms of social anxiety as this abuse determines traumatic memories that influence the current vision of self as central to one's identity (Shahar et al., 2015). As such, it appears that the experience of shame is central to social anxiety. The authors point out that one possible explanation is the most direct link between emotional abuse and shame.

Shame and its consequent defenses can produce various outcomes. To illustrate, if the general affect of shame is a desire to hide, as with psychosis and suicide, that “disappearance” can be even more frequently obtained by modifying character traits (and is consequently personological), as the subject remains constantly on guard against any experience of shame. At other times, reactive formation is not implemented toward the drives, but instead toward the anguish of shame, resulting in shamelessness (Levin and Wurmser, 1996). The lack of this affect is present both in some psychotic constellations and in psychopathy which is characterized by an “absence of shame”. In fact, Wurmser argues that alexithymia, a symptom often present in schizophrenia, represents a character defense against exposure that can induce shame through inhibition, denial, and reactive formation (Levin and Wurmser, 1996). This seems to generate an impossibility of communication dominated by the desire for fusion impregnated with omnipotence.

Below, we aim to illustrate the hypotheses on the origins of shame from various theoretical perspectives. The parallels in the interpretation of this affect both among different disciplines and within conceptions in the same context, for example, classical psychoanalysis and Self-psychology. Various disorders characterized by shame are then taken into consideration, highlighting the different expressions of this affect both in a qualitative and quantitative sense.

## Shame in psychoanalysis, philosophy, and psychopathology

For Kohut, shame can paradoxically represent “a normal disturbance” resulting from an obstacle to the regular flow of exhibitionistic libido. Specifically, shame emerges from the failure of relational experiences from which self-esteem and exhibitionistic pride should be obtained (Kohut, 1997). Meanwhile, other authors affirm a significant relationship between shame and being ignored or neglected by others (Zahavi, 2014). Similarly, Zahavi (2014) notes that “[i]n recent psychoanalytic theorizing, it has been proposed that shame is a reaction to the absence of approving reciprocity. If so, it would situate shame right at the core of our interpersonal life”. Zahavi agrees that already in primary development, the sense of themselves and the relative emotions reveal the exposed and interpersonal nature of the self; they are regulated by the visibility of the self as an object of the other's attention and evaluation. “To that extent, the evaluating perspective of others may play a role in the structure of the emotion even if they are not factually present or explicitly imagined” (Zahavi, 2014).

Zahavi also notes that Reddy's observation, about the early awareness of children regarding the attention paid to them, seems to be placed in this trajectory, while that inherent in the attention of others directed toward other things in the world only occurs at a later time. The first, however, represents one of the most powerful human experiences. Moreover, similar to Sartre, Zahavi (2014) claims that low self-esteem generates shame as a function of how much it affects and alters relationships and connectedness. The frequent coexistence between low self-esteem and shame is important because, in the face of the relational origin of shame, self-devaluation acquires more the meaning of a limit of acceptance and closeness than a disappointment with respect to one's own higher standard. This relational element and connectedness appear to be a consequence of the fundamental and precocious human need to belong. The corollary of this link can be seen in the idea of Pardede et al., that human beings have a burning need for belongingness and to be accepted for themselves and not based on a socially-presented Self. In other words, rejection and emotional isolation from a social context can be experienced when one is not able to truly be oneself (Pardede et al., 2021).

It seems useful to note the various proposals for articulating this fundamental human need, as well as to explore the direct and inverse proportionality between dimensions. Two dimensions that have been identified in the aforementioned basic need, are belongingness and acceptance. Different theoretical models have been hypothesized with regard to these dimensions, although they have not yet been confirmed in experimental studies. The three factors that can be identified separately are “belongingness”, “emotion acceptance” and “self-representation”. The first, “belongingness”, is in line with Kohut's specular need, i.e., the feeling of connectedness in relation to one's social relationships and alikeness in being part of the “other”. The second, “emotion-acceptance”, concerns the self-permission to feel one's emotions and to express them to others, with an open acceptance. In other words, emotionally experiencing events openly, without defense, regardless of whether they are positive or negative. The third, “social self-representation”, is the need to represent oneself as a person of worth. As underlined by Cooley and Kohut, people try to portray themselves as someone to be liked and valued (Pardede et al., 2021).

There is a significant positive relationship between “belongingness” with both “emotion acceptance” and “social self-representation” and, to the contrary, a significant negative relationship between “emotion acceptance” and “social self-representation”. The first correlation means that the more one needs to belong, the more one feels the need to be accepted for one's emotions and to be allowed to share them with others. Thus, the way one presents oneself socially must be involved in fulfilling the need for belongingness. The parallel between belongingness and emotion acceptance is in line with the viewpoints of Lee and Robbins, that the need to belong is fulfilled through emotion-sharing and reciprocal connectedness. The latter shows that the greater the need to be accepted openly for one's emotions, the less the need to present oneself in a favorable view in order to receive respect, and vice versa. The feeling of belonging as a whole appears in line with the twin need, fundamental in Kohutian theory, which is expressed in experiencing the connection and sharing of similarities with others (Pardede et al., 2021). Similarly, Nathanon's statement, in the context of the aforementioned needs, argues that “shame-humiliation is an affect program designed to be triggered in situations when there is an impediment to the continuation of positive affect but the person remains interested and desirous of the thing that produces positive affects” (Gilbert and Andrews, 1998). After all, Freud considers shame as a defense against the voyeuristic drive which, for the father of psychoanalysis, also encompassed exhibitionism and self-exposure. Consequently, Nunberg affirms that “shame is a reactive formation of the Ego to the desire to exhibit” (Levin and Wurmser, 1996).

In this direction lies the theory that the parasympathetic-demobilization response of very young infants is elicited, for example, when the mother fails to mirror facial expressions (Gilbert and Andrews, 1998). It is the prototype of the shame affect: some theorists come close to suggesting that the parasympathetic response associated with reduced arousal is what shame is (Gilbert and Andrews, 1998). An interesting question is whether shame is triggered by a narcissistic lack that relates to the grandiosity or the consequent failure of a need for closeness, a corollary of the anguish of disintegration, which is the fundamental anguish for Kohut. In this regard, Wurmser's reading of the anguish of shame is noteworthy, as a different version of the anguish of separation in which there is the fear of losing the object of love and with it, the fear of the loss of the Self: “one who is not loved, stops loving himself, with the feeling of being frozen” (Levin and Wurmser, 1996). This freezing can also lead to disorganization and afinalism, as highlighted in the Beebe and Stern experiment described below (Lichtenberg et al., 2012).

Shame seems to be the affect that most viscerally involves the body. It is intriguing to note the profound link that Sartre outlines between shame, the gaze, and the body which seems to be in line with the significant presence of shame in psychic disorders that primarily involve the body dimension (such as eating disorders, and others stemming from physical and sexual abuse). Wurmser states that only conflicting perceptions and forms of self-exposure trigger the reactive model of shame, differentiating it from signal anxiety. Owning and showing parts of one's body or emotions is not shameful in itself, but losing control over feelings and showing them to others can induce shame (Levin and Wurmser, 1996). One could question the relationship between this loss of control and the fear of abandonment by the object. After all, Kohut considers shame and aggression to be secondary to unsatisfied narcissistic needs (Kohut, 1980). Retzinger's statement, that shame-anger arises from threats to a social bond—in particular, attachment bonds—seems to go in this direction. A similar view was held by Lewis, who suggested that shame-anger was a kind of protest-anger related to the breaking of a bond (Lewis, 1992).

In fact, the level of shame seems to represent the balance between inner loneliness and the availability of self-objects. Piers links shame to narcissism in that shame derives from a tension between the Ego and the Ideal Ego (Chasseguet-Smirgel, 1976). The Ideal Ego is a hinge concept between absolute narcissism and objectivity. The projection of infantile narcissism on the object is evolutionary toward reality and objectivity; the Ideal Ego implies the idea of a project. The project is strictly correlated to maternal care. To illustrate, in some mammals, a member of a litter that has not been licked by the mother dies, and when the mother has forgotten to lick the genitals of her young, they do not urinate (Chasseguet-Smirgel, 1976). Shame seems to be closely related to the lack of projectional design.

The link between the Ideal Ego and original fusion is underlined in Freudian thought. Chasseguet-Smirgel (1976) notes that for Freud, the Ideal Ego is “the substitute for the lost narcissism of childhood”. Nunberg notes that the Ego while obeying the Super-Ego for fear of punishment, submits to the Ideal Ego for love (Levin and Wurmser, 1996). Man is thus a sick animal in search of a bygone time, such as a return to the mother's breast, the one in which he constitutes his own ideal (Chasseguet-Smirgel, 1976). After the rupture of the primary fusion, there is the projection onto the object, the child's first Ideal Ego of the torn narcissistic omnipotence that he or she will always strive to regain (Chasseguet-Smirgel, 1976). In 1914, Freud stated that being loved increases self-esteem: “[b]eing loved facilitates the realization of the Ideal Ego. The loss of the love of society would consequently lead to an enlargement of the gap between the Ego and the Ideal Ego and would be the equivalent of a narcissistic wound. The loss of love, especially when love is a narcissistic food, can lead to very particular effects related to shame” (Freud, 1980).

Indeed, we can observe that exhibitionism connected with a specular need seems to contain a need for fusion. In Kohutian terms, perhaps we could speak less of a perfect self-image and more of a basic element such as the recognition of one's existence through the sense of belonging to the human body (Kohut, 1980). As Wurmser notes, the threat inherent in the anguish of shame is abandonment (Levin and Wurmser, 1996). He states that “if we compare the ‘image of the Ideal Self' in shame with the ‘code of ideal actions' in the sense of guilt, we realize that the former is fundamentally more archaic, more comprehensive, and less differentiated than the latter” (Levin and Wurmser, 1996). Moreover, it would seem that a link between omnipotence and shame can be drawn as potential consequences of the unfulfilled mirror and twin needs.

In a Lacanian perspective, anguish arises when the object of enjoyment, a real object, is found to be in immediate relationship with the subject, that is, in the absence of mediation on the part of the “symbolic” (real object as unspeakable, not caught in a network of signifiers) (Lacan, 2007a) or part of the “imaginary” (real object insofar as it is not mirrorable) (Miller, 2006). Psychosis teaches us something about non-mediated, direct relationships. In particular, this is evident at the moment in which the anguish and bewilderment that precede the delusional construction veils the unspeakable object, and appeases the anguish itself. This delusional construction appears to be a symbolic-imaginary one that produces an object, the object of delusion, speakable and mirrorable. Therefore, if anguish arises when the object of enjoyment is in a non-mediated relationship with the subject, the arising of the shame affect, in front of the other, signals the presence of a “modest” relationship of the subject with his own object of enjoyment. This modest, or we could say veiled, relationship is itself symbolic in nature as it is situated at the level of the cultural norms that regulate a given social functioning.

## Clinical case study

A lack of modesty contrasts with an excess of shame often present in neuroses where there seems to be a significant connection with abandonment. This is illustrated in the following clinical case study in which the connection between a significant experience of shame and maternal abandonment is represented in the symptoms of trichotillomania.

A 41-year-old patient, M, suffering from major depressive disorder and trichotillomania undertook group psychoanalytic psychotherapy. The symptom of trichotillomania started during puberty (coinciding with menarche) when she also wanted her breasts not to grow. As an adult, the patient, who wore a well-finished blonde wig, kept silent in the group for many months about her trichotillomania and the consequent alopecia for which she felt intense shame. In fact, M spent years pulling her hair out in secret. Another highlight of the patient's story appeared in later years when she was pregnant and said that she would kill herself if her son were to see her without hair. Eventually, the trichotillomania regressed completely before the baby was born (Saya et al., 2018).

Klein (2006), in 1952, observed that children often reassure themselves of a good relationship with their mother by playing with her hair before breastfeeding. Indeed, M later associated the urge to pull her hair out with feelings of loss and abandonment by her mother. For M, her alopecia seemed to represent maternal abandonment while the wig represented a false fusion. Her intense shame could be attributed to the meaning of the symptom: the experience of maternal abandonment and its fusional pseudo-repair with fake and idealized hair (i.e., the wig). Another element was that, as a child, the patient experienced, with great suffering, the evidence that the blonde shade of her natural hair was slightly darker than that of her mother.

It appeared difficult for the patient to access her position as wife and mother. A veiling of enjoyment emerged, inherent in the symptom (trichotillomania), in the gaze of the other. Freud (1984) considered the symptom to be a substitute for satisfaction that implies repression. The aforementioned link between trichotillomania and feelings of loss and abandonment by her mother shows a truth discovered by the patient, a truth of a phantom order, which is a position of enjoyment with respect to the “maternal other” that of being abandoned by her mother. The patient was able to speak without shame about her abandonment because in that relationship she saw herself as a passive element. The truth of this phantasmatic position came to us from the repetition of the same: she found herself in various situations in which she complained of being abandoned by the other, but she did not take on an active role in her abandonment. The stakes of the game are veiled.

## Shame and psychosis

In psychosis, both psychopathology and psychoanalytic theories underline the links, respectively, between an excess of shame and the so-called negative symptoms and the defensive absence of conscious shame (corresponding to a gigantic share of unconscious shame) in positive symptoms. With regard to the two poles of the shame continuum, Wurmser notes that at the other end of the continuum of anguish signals there is an anguish of shame that results in a symbolic death in which inhibition can appear in a form of a non-specific block of all feelings, actions, and perceptions (Levin and Wurmser, 1996). In this case, where the regulating role of relationships is lacking, it becomes a totalizing defense against affects, a probable corollary of the panic experienced in a situation of rejection and abandonment (fairly typical of psychosis), different from a very clear awareness of the potential danger of suffering a refusal often characteristically present in neurosis (Levin and Wurmser, 1996). The function of shame as the modulator in interpersonal relationships would fail either by generating a form of withdrawal, probably assimilable to negative symptoms, or by causing shamelessness, often evident in positive symptoms.

Piers and Singer affirm that “behind the feeling of shame lies not the fear of hatred but the fear of contempt” (Levin and Wurmser, 1996). The contempt for the other has changed the person into nothing. In this extreme form, the contempt for the individuality of the person leads to the schizophrenic's condition of total loneliness (Levin and Wurmser, 1996). The loneliness of the schizophrenic could perhaps be seen as the impossibility of the experience of exhibitionism, including the subliminal signals of shame, which contains the hope of mirroring and therefore in the denial of the tension toward the object (from a Kohutian perspective, the research of a self-object, albeit archaic). In such a context, the specular need would contain the desire for the relationship as the need to exhibit good aspects of oneself would be instrumental to being accepted. In psychosis, there seems to be the transformation of the shame, generated by the rejection of the other, that in neurosis becomes something different: the subject no longer knows shame because he has become ashamed (Termini, 2018). The experience of being the object of contempt can probably be connected with the etiopathogenetic theory of schizophrenia relating to the absence of the necessary symbiotic phase between mother and infant. Interestingly, in this regard, the etiopathogenetic theory that identifies the cause of schizophrenia in the impossibility of mother-child symbiosis perhaps could represent a proto-experience of a mirroring deficit. The loss of love that is recorded in shame is a form of total indifference toward oneself with one's rights and prestige (Levin and Wurmser, 1996). In this context, there is a link between shame and feelings of non-existence. Perhaps extreme forms of loneliness resulting from the absence of significant introjects in the internal world exclude the role of modulators of interpersonal relations. Extreme modesty corresponds to an object's lived experience, such as an invader who emphasizes a desert-like lack of the other, while shame, usually unconscious, pertains to inner loneliness. In this case, the presence of extreme modesty, a counterpoint to a significant amount of unconscious shame, seems to connote the negative symptomatology of schizophrenia. Omnipotence, in this context, appears to constitute the defense against self-contempt on the part of oneself and others. This defense can generate positive symptoms, such as delusions of grandeur and/or of persecutors, as well as hearing voices in dialogue and/or voices that comment on the patient's acts.

There is a long psychiatric speculative tradition in which shame has been considered a sort of threshold on the psychotic path, as shame's evolution can often include guilt or persecution. Kohut argues that shame cannot be processed but can only be expelled by a “magical” annulment or by being turned into anger. The Kleinian theory that refers to schizophrenia as being due to a high presence of envy, seems also to point in this direction. Moreover, the lack of proper mother-child symbiosis represents the trigger of an overbearing level of anger.

In schizophrenia, a psychic event triggered by shame regressively generates an expression of the primary thought process. After an experience of shame, the boundaries of the self are weakened and one's perspective becomes unrealistic and undifferentiated (Keen et al., 2017). The experience of shame is linked to a kind of sinking within oneself, into an anguished feeling of transparency. In this regard, the studies on the pre-psychotic phase of schizophrenia, characterized by transitivism and appersonation, are interesting (Saya, 1988). The primary process, triggered by shame, seems to conceal this affect in parallel with the dilution of the secondary process. Since it is known that schizophrenics are capable of a secondary process, the emergence of a primary process can specifically indicate that an event of shame (or fear of shame) is at work (Keen et al., 2017). This would seem to be in line with Morrison's statement that identifies the emergence of the primary schizophrenic process as an indicator of an experience of shame. “A long psychiatric speculative tradition, ranging from Monkowski to Meissner, considers delusion as triggered by a self-inflicted wound” (Ballerini and Rossi Monti, 2011). Kretschmer believed that unbearable feelings of shame resulting from a narcissistic injury were the core of the delusional turn. Along these lines is Meissner's assertion that humiliation represents the most explicit correlation of that crisis of the Self that is expressed in the “devastation” produced by the schizophrenic process that activates persecutory symptoms (Ballerini and Rossi Monti, 2011). This wound of the Self corresponds to events that resonate with personological aspects on which the modulation of self-esteem seems to depend so that the feelings of shame would lead to identity problems.

In many kinds of delusions, the exuberant sthenic defense signals the sensitivity of the asthenic part while the original shame and wound appear as a pale reflection. The substantial disappearance of conscious shame in the schizophrenic process appears in both paranoid and in catatonic patients, in what Morrison labels as “bypassed shame”. Castelfranchi considers that “bypassed shame” functions as a type of protection of the self-image, as it allows the individual to maintain the sovereignty of feeling “adopted” (Ballerini and Rossi Monti, 2011). This sovereignty would seem in line with the main corollary of shame, the need to belong. The disappearance of conscious shame also represents the outcome described by Fenichel as a counter-phobic attitude toward shame (Fenichel, 1951). In this regard, Wurmser's reading of the anguish of shame as a different version of the anguish of separation in which there is a fear of losing the object of love, and with it the fear of the loss of the Self, is explanatory: one who is not loved stops loving himself (Levin and Wurmser, 1996).

Similarly, for infants, the affects that accompany aversive withdrawal responses are discomfort, fear, shame, and low adaptability. The primary constructive strength of the innate patterns of the early childhood aversive motivational system depends on the quality of the caregiver's reparative responses to these cues (Lichtenberg et al., 2012). If the aversive motivational system organizes itself and becomes dominant early in childhood, children will tend to states of prolonged antagonism and withdrawal. Instead of looking for the positive effects of the relief of suffering that the positive response to anguish signals provide, they tend to derive a sense of security from the familiarity of anger, discomfort, pain, disgust, or shame (Lichtenberg et al., 2012). This, in addition to being able to connect with psychotic symptoms, seems to represent an important component of moral masochism. In this regard, Wurmser's statement that when affects such as tenderness, love, sweetness, and dependence evoke a sense of deficiency, they generate shame, and therefore must be kept hidden both from others and even from oneself, is interesting (Levin and Wurmser, 1996). This concealment is often reinforced by reactive formation, generating masochistic pseudo-aggression.

Likewise, we can place Wurmser's affirmation of the frequent presence of the sense of guilt as a defense against shame and the qualities of hatred and anger directed toward the Self which constitute defenses against the more terrifying affect of self-contempt. The sense of guilt, hatred, and anger, represent, albeit negatively, a centrality of oneself as opposed to concealment that contains the idea of exclusion and ultimately of non-existence (Levin and Wurmser, 1996). Such dynamics can also lead to disorganization and afinalism, as highlighted in the Beebe and Stern experiment. Mothers approached their 6-week-old babies, lying down in a quiet state, placing their faces in the children's field of vision. The children responded with increasing full-body arousal. If the mothers, following instructions, kept their faces expressionless and remained silent, the babies initially increased their movements and vocalizations in response. As the mothers continued not to respond, the children's efforts became more and more frantic and disorganized (Lichtenberg et al., 2012). Perhaps this reaction could be considered a harbinger of shame as the child's need for specularity is expressed through a display of negative aspects. Other experiments similar to this are cited in the literature and are always carried out in early childhood, like that of the still-face experiment in which there is an alternation between the still-face episode and normal face-to-face interactions. This produces attempts to re-engage the gaze and then often leads the avoidance of eye-contact (Zahavi, 2014). “The infants have expectations about the way face-to-face interactions should proceed, and about the nature of appropriate interactive responses from social partners” (Zahavi, 2014).

Binswanger in “[T]he problem of shame and the schizophrenic process” identifies the core of the schizophrenic process, activated by shame, in the transformation of the human being in the sense of a loss of freedom of the fluid displacement of the “inner limit of sin” no longer established by the Self but by the social environment. This results in a permanently established limit without the desirable modulation of the cause and the level of shame (Ballerini and Rossi Monti, 2011). The center of gravity of one's existence is shifted from one's Self to the judgment of others, experienced as fixed with the consequent feeling of personal emptiness (Ballerini and Rossi Monti, 2011). This seems to coincide in part with the Kohutian idea regarding individuals who need absolute control over an archaic environment as the maintenance of their self-esteem depends on the unconditional availability of the appropriate specular self-object and the idealized object that allows the fusion (Kohut, 1984).

In psychosis, primitive stages, such as a child's normal grandiose stage, are bizarrely achieved with a psychotic, disturbed, and detached grandiosity. Primitive experiences of fusion are psychologically reconstructed as influencing forces with hostile intent (Kohut, 1997). If shame represents psychic pain with respect to a rejected exhibitionism, the persecutory symptomatology could be read as an emphatic and shameless pathology of exhibitionism.

By the same token, the commenting voices heard by schizophrenics seem to translate the externalization of the observer who has the task of returning the image of his Ego (and in which he sought a narcissistic confirmation aimed at bringing his Ego closer to the ideal) to the object. This role, as Freud (1980) said in 1914, is first entrusted to parents, and is later carried out by “peers”. Therefore, this symptom could be read as a pathology of normal exhibitionism. The peculiar role of the body could account for the physical neglect and perception disturbances of schizophrenics.

With regard to the negative symptoms of schizophrenia, these may also refer to a “proto-defense” described by Wurmser: an avoidance reaction in the form of hiding and blocking perceptual activities, a very archaic form of defensive restriction with respect to instinctual greed. This could result in a form of “freezing”, in the form of paralysis and stupor, or a non-specific blockage of all feelings, actions, and perceptions (Levin and Wurmser, 1996). In this context, alexithymia can be inserted as a character defense against shame-inducing exposure.

## Shame and depression

Shame and some types of consequent defenses seem to play a significant role in depressive symptoms. The above-mentioned “proto-defense”, which aims to make the shame disappear, in its most radical form, leads to suicide, which is typical of depression. This is in line with Freud's view of shame as a form of resistance to libido. In some cases, the anguish of shame arises when the object of enjoyment is in immediate relationship with the subject, in the absence or with minimal mediation on the part of the symbolic: this could be linked to anhedonia. In its extreme form, contempt leads to loneliness in schizophrenia. Perhaps this feeling can be compared to a depressive's sense of loneliness due to the lack of an internal mother. If one fails to achieve the beloved and desired ideal, an emotion similar to nostalgia is felt. In depression there is a renunciation of the desire to show off, to do something to please others and therefore also oneself. In this regard, Wurmser's observation, of the significant ambivalence in relationships that induce shame, is noteworthy. The depressive renunciation seems to be permeated by the defense of introjection also regarding the shame, induced by the conflictual relationship with the object.

It can be noted that when the asthenic aspect is not covered by the sthenic defensive counterpart, one experiences bewilderment, similar to pre-melancholic desperation, which consists of the desire to be in two places at the same time, enveloped in doubt. The essential aspect of shame is wanting not to be present, to disappear: neither to see nor to be seen (Ballerini and Rossi Monti, 2011). The contiguity between the area of shame and pre-melancholic despair represents the central point of the narcissistic foundation of existence. From the crisis of the latter, the melancholic and/or paranoid paths begin (Ballerini and Rossi Monti, 2011). A significant difference between the aforementioned paths consists in the fact that in the melancholic ones there is a more explicit involvement of the body which in this case shame immediately involves the body in all its objective-subjective aspects.

Shame's attack on the self easily becomes a circularity of hostility or anger toward oneself and/or others. Corollaries of unconscious shame include a tendency to doubt oneself and one's abilities, feelings of hopelessness, and a tendency to abandon situations. Personalities with an excessive tendency toward shame often exhibit social anxiety and phobic fearfulness, lack self-confidence, experience extreme shyness, worry about personal inadequacies, hold self-derogatory attitudes, feel frequent depression, and shrink away from any risk-taking assertiveness, or public exposure that may bring on critical confrontation with or ridicule from others (Gilbert and Andrews, 1998). However, it should be noted that this profile has large areas of overlap with depressive disorders. Wurmser asserts that it is the frequent presence of the sense of guilt as a defense against shame and the qualities of hatred and anger directed toward the Self that constitute defenses against the more terrifying affect of self-contempt (Levin and Wurmser, 1996). Therefore, the core of depression, represented by the sense of guilt and the turning of anger toward oneself, derives from defenses against shame.

## Shame and eating disorders

Pathological shame is considerably present in eating disorders, especially in bulimia (Gilbert and Andrews, 1998). The relationship between shame and eating disorders has been known since early case descriptions of those who suffer from them. One could ruminate on the overwhelming presence of shame in psychic disorders that primarily involve the body dimension (such as eating disorders or physical and sexual abuse). Many have studied the subject of the gaze of the other from a Lacanian viewpoint, finding that it is a fundamental element of the psychopathology of anorexia. In the same vein, according to Sartre “[t]he gaze of the other, real or imaginary, is what makes me realize that: (1) I have a body; (2) I am vulnerable and I can be hurt; and (3) I occupy a space (I cannot disappear, but at least I can hide) and a defined time. This represents the irreducible human condition of being seen: I am seen by the other because I am a body”. Sartre also states that the body is all psychic. This concept is in line with Plato's statement that defines the body as what individualizes the soul, noting that “the encounter with the other is above all an encounter between two bodies, two things separated by nothing” (Sartre, 2014).

What is more, Wurmser states that only the perceptions and forms of self-exposure that are conflicting trigger the reactive model of shame, differentiating it from signal anxiety. Owning and showing parts of one's body or emotions is not shameful in itself, but the feeling of shame comes from such exhibitionism when it is linked with losing control (Levin and Wurmser, 1996). One might wonder if losing control is correlated with the fear of abandonment by the object.

Recent studies have found an association between the experience of body shame and eating disorders. Many studies have indicated that a significant proportion of adolescent girls are engaged in disordered eating behaviors and unhealthy weight reduction practices, such as dieting, vomiting, over-exercising, or inappropriate use of laxatives and diuretics (Mustapic et al., 2015). Body shame involves an emotional component in the negative evaluations of one's body, which includes a desire to hide oneself. This need to hide often leads to the concealment or stylization of the body through obesity or anorexia, respectively.

Body shame pertains to negative feelings about the self in general and mediates the relationship between body dissatisfaction and eating behaviors, particularly for adolescent girls. Body shame and body dissatisfaction explained statistically significant amounts of variance in eating behaviors while controlling for age and BMI. Body shame highlights girls' failure to attain the unrealistic ideal body despite their best efforts to do so. The evidence of this study is also that the distinction between their physical appearance, self-worth, and disordered eating behaviors is blurred (Mustapic et al., 2015).

Another key point is that shame proneness can be an important component for the development and maintenance of eating disorders due to a strong correlation not only with symptoms of eating disorders but also with the psychological aspects of these. The authors consider these disorders, in both clinical and non-clinical samples. In this study (Cavalera et al., 2016), the Eating Disorder Inventory 3 (EDI-3) and the shame proneness subscale of the Italian version of the Test of Self-Conscious Affect (TOSCA) were administered to investigate various psychological dimensions such as self-esteem, insecurity, emotional instability, and perfectionism. Shame proneness was higher for the clinical group than for the non-clinical group. Higher shame proneness reported in TOSCA for the clinical group evidenced a higher personal predisposition regarding negative global attribution toward a defective self that can trigger dysfunctional eating behaviors. In the non-clinical group, all the eating disorder symptoms and all the psychological variables showed a strong correlation with shame proneness. In the non-clinical population, high scores on most of these variables were usually related to a significant risk of eating disorders.

Considering the general clinical group, almost all of the psychological EDI-3 primary scales show significant correlations with shame proneness. First, shame proneness is strongly related to the general psychological maladjustment variable: higher scores in this composite scale indicate the presence of psychological maladjustment and suggest dysfunction in both personal and interpersonal psychological domains. Consistent with the previous findings, shame proneness correlated with low self-esteem and ineffectiveness in the general clinical group. One explanation for this association suggests that eating-disordered individuals with low self-esteem and ineffectiveness could try to cope with negative events through emotional eating behaviors.

Interestingly, although in all three sub-clinical groups (anorexia, bulimia, and binge-eating) shame proneness showed strong correlations with interoceptive deficits, this relation is particularly strong in the anorexia subgroup. For these patients, the tendency to experience pervasive feelings of worthlessness elicited by shame proneness was correlated with greater difficulties in consciously perceiving signals arising from the body. This link seems to be crucial to understanding the processes related to starvation and food denial typical of anorexic patients. Similarly, the anorexia subgroup showed the strongest association between shame proneness and perfectionism. Defensive shame-based blame and anger may subsequently lead either to withdrawal (by either or both parties) or to escalating antagonism, blame, and counter-blame, and this negative pattern could be particularly pervasive for binge-eating and bulimic patients. Therefore, shame-proneness is associated with many psychological aspects related to very different domains of eating disorders (Cavalera et al., 2016).

## Shame and personality disorders

Miller suggests that personality itself—particularly obsession and narcissism—can be built around scripts about what is and what is not shaming and defending against shame (Gilbert and Andrews, 1998). In various personality disorders, a tendency toward antagonism, withdrawal, or alternation between these two poles, derived from the self-organization of the aversive motivational system, can be highlighted. The persistence of the affects present in the aversive motivational system, such as anger, pain, and shame itself shows how the latter paradoxically gives greater security regarding the bond. This, in addition to being connected to psychotic symptoms, can represent an important component of moral masochism which, according to Bergler (1978), constitutes the basic neurosis. For Piers and Singer, as cited by Wurmser, there is a secret power in narcissistic maneuvers that prevents the possibility of suffering defeat by others by pre-emptively inflicting it on themselves, as a form of internalized shame consisting of an active and constant attempt to obtain punishment, through a series of particularly clumsy actions (Levin and Wurmser, 1996).

Shame and its defenses are symptoms present in various personality disorders. Some authors have highlighted how shame is the nuclear element of borderline personality disorder, noting the presence of shame as more significant in borderline personality disorder than in controls. This result has been linked to the association between trauma and shame already highlighted by Buchman-Wildbaum et al. (2021). Significantly frequent sexual abuse, mistreatment, and neglect suffered by borderlines in childhood often produce feelings of shame (Buchman-Wildbaum et al., 2021).

The Psychology of the Self has specifically highlighted the role of shame in narcissistic disorder. With regard to narcissistic personality disorder, Kohut (1984) identifies individuals in whom the maintenance of self-esteem, and therefore of the Self, depends on the unconditional availability of the appropriate specular self-object and the idealized object that allows fusion with the consequent need for absolute control over the archaic environment. Kohut (1982) blames intense shame and anger on the unexpected lack of cooperation by the mirror object—both the external environment and the internal structure of the superego have incorporated the approval function of the archaic environment. This occurs, above all, when the grandiose-exhibitionistic archaic Self floods the Ego's reality with an exhibitionistic investment that is not neutralized, overcoming the neutralizing powers of the Ego. In this regard, it is useful to underline the intimate intertwining between shame and secondary anger, both reactions to a narcissistic wound. Moreover, schizoid and avoidant disorders differently show the, respectively, unconscious and conscious declination of shame with the consequent dichotomy inherent in conscious desire with respect to interpersonal relationships.

## Shame and COVID-19

Shame may underlie the development of psychiatric co-morbidities in organic diseases. As recently highlighted during the COVID-19 pandemic, the presence of a contagious organic disease (involving the community, and therefore correlated with a high social stigma) has, for some, generated long-lasting feelings of shame with consequent risks to mental health. The persistence of feelings of shame over time may have contributed significantly to the onset of frank psychiatric disorders such as eating disorders, anxiety, depression, and self-injurious and suicidal behaviors (Cavalera, 2020). The increased incidence of such mental pathologies has been observed in the course of previous disasters and, in this light, further studies between trauma and shame would be interesting. In some people infected with COVID-19, this experience may have arisen from feeling defective and helpless. The shame related to COVID-19 induced traumatic aspects such as feelings of worthlessness, inferiority, and helplessness that extended to the entire Self (Cavalera, 2020). Therefore, shame, exacerbating a negative global attribution, is associated with negative effects on mental wellbeing. The experience of shame may have been so pervasive that it led some people to hide clinical news from health professionals, thus increasing the risk of infection. Even the prolongation of situations of social withdrawal over time is related to the prevalence of this emotion. Furthermore, the dimension of guilt linked above all to the fear of infecting others was very present. The strong social stigma toward health workers was important and led to an increase in self-injurious and suicidal gestures (Cavalera, 2020).

## Discussion

There is a noteworthy difference between western and eastern perspectives regarding shame which reflects the significant gap between individualistic and collective societies where spirituality and the concept of duties toward the group play different roles. It seems that in the west the valence of shame results in a heterogeneous spectrum in which its pathological declination in various disorders is emphasized (Gilbert and Andrews, 1998).

Lewis describes shame as “a strongly negative and painful state that also determines the interruption of ongoing behavior…” (Bhushan et al., 2020a). On the other hand, shame in Eastern societies, appears to constitute an exclusively positive psychological construct as it represents a reflective process of self-evaluation that helps one follow the path outlined in the scriptures and belongs to noble people (Bhawuk, 2017). In Sanskrit the term lajjA summarizes the concepts of shame and guilt and represent a delicate transitory emotion, whereas these concepts are distinct in Western culture. In fact, from the eastern point of view, such a differentiation between the cultures of shame and guilt must be questioned. LajjA represents the most important virtues which guide one at the various stages of life, as a kind of inner ruler indicating appropriate actions to take and helping one to avoid inappropriate ones, thus mediating between desire and action as an internal (negative feeling) and external (judgment of others) preventive mechanism. Considering lajjA in the context of an “inner guide”, it is notable that in Indian mythology, LajjA is the name of the wife of Dharma, the God of righteousness (Bhawuk, 2017).

With that in mind it is interesting to consider that although everyone can be tempted at times to neglect sacrifices, charity, austerity or appropriate actions, lajjA becomes the barrier that prevents one from neglecting one's duties. It therefore has an important ethical function as an interior impediment guided by scriptures and cultural norms of appropriateness that lead to noble behavior. As such, it represents a healthy emotion and an antidote to anger and other negative emotions. Thus, without lajjA, knowledge and wealth cannot be used properly and therefore cannot be in the path to happiness (Bhawuk, 2017). From this point of view, shamelessness seems to coincide with an unethical, amoral conduct. This element, which in Western individualistic society may be implicitly functional to individual success, in Eastern society has a completely negative connotation, as unethical behavior would coincide with the impossibility of walking the path to happiness.

Another interesting debate concern the differentiation between guilt and shame in eastern and western cultures, in particular the concept of qualifying guilt and shame on the basis of the public or private nature of the situation that elicits the emotion. According to the article by Bhusan, Basu, and Dutta, “[w]hile shame arises out of publicly being exposed for something that is disapproved of by others, guilt is a private emotion that is self-generated out of one's conscience” (Bhushan et al., 2020a). They also note that others believe that guilt and shame are both public “as the transgression or failure is known to people” (Bhushan et al., 2020a). It is argued that the focus on either the Self or one's behavior can also be used to differentiate shame from guilt, as focusing on the Self leads to shame (which is considered a positive emotion in eastern cultures) and focusing on behavior leads to guilt which compels reparation. To illustrate the idea of shame being a positive emotion, Bhusan quotes Sibia and Misra who submit that “to experience lajja is to experience [a] sense of graceful submission and [a] virtuous, courteous well-mannered self” (Bhawuk, 2017). The study by Bhusan, Basu, and Dutta highlights the significance of reparation in the experience of guilt while the absence of the reparative purpose results in shame and remorse. The lack of restorative purpose in shame would seem to be due to its predominantly preventive function. As in “cultures of shame”, observance of the rules is achieved through the proposition of positive models of behavior and those who do not adapt to these models incur social blame (shame in an objective sense) and a subjective feeling of inadequacy, in its own right defined as shame (Bhushan et al., 2020a).

Differently from the moral analysis of shame and guilt seen in the study by Bhusan, Basu, and Dutta et al., another study, conducted by Bhushan et al. (2020b) focused on the different physical manifestations of guilt, shame, and remorse. Specifically, the variations in temperature due to increased blood flow in different regions of the face related to those emotions. This differentiation was partial as thermal increases were found in specific areas with various overlaps (Bhushan et al., 2020b). There is a logical basis for studying the physiological aspects of the facial region related to guilt and shame as blushing is a common reaction to shame and guilt. This universal response is linked to various psychological concepts such as losing or saving face. For example, in Chinese culture, one's moral conduct and social image constitute the primary source of identity where shame relates to the fear of “losing face” and therefore represents the main regulator of social life and respect for cultural norms and moral values. A corollary of this in Indian culture is Krishna's statement that “Infamy is worse than death for noble people”. According to Bhawuk (2017), in Indian culture, embarrassment is to be avoided at all costs and this desire is related to the concept of saving face in China and Japan. It follows that it is shame, more than a sense of guilt, that represents the moral emotion that has the function of pushing people to conduct themselves in harmony with the social order (Mazzei and Volpi, 2010).

In 1946, the American anthropologist, Ruth Benedict, defined Japanese culture as a “culture of shame” as the sense of shame is instilled in the Japanese from childhood, in accordance with the belief that the group comes before the individual. Showing society that you are able to feel ashamed for illegal actions, or for what you are accused of, counts toward social acceptance and, consequently, also affects career prospects (Benedict, 2009).

The purely relational meaning of shame is often highlighted by authors as shame seems to be activated by an innate need for relationships and the sense of belongingness that is expressed therein. The experience of the transitory state of shame, hence a transitory affect, does not indicate the presence of any psychic disorder but, on the contrary, facilitates the rhythm of relationships and consequently the social and personal adaptations within a group, thereby maintaining one's protection through the association with that group.

Pathological shame is an almost ubiquitous affect of mental disorders. In the various disorders, this affect can take on significant quantitative and qualitative differences and therefore declines in an extremely heterogeneous way. This affect, both in its physiological and pathological forms, seems in fact to pursue a protective intent in encounters with the other. In the first form, this aim is to modulate the rhythm of the interactions with the other, becoming the guarantor of belonging to a group. In the second form, there seems to be a sort of surrender to the refusal of the other, whose gaze is fleeting, consequent to abandonment anguish. The intimate link between shame and the need to belong shows its exquisitely relational origin. The diverse models for needing to belong seem to find a correspondence with the primary needs highlighted by Kohut. “All emotions are self-involving in some sense and they imply a relation between self and object and often a triangular relation that also includes others” (Kohut, 1997).

An important question that runs through both philosophical and psycho-dynamic literature regards the presence and role of the other in the genesis of shame. Another reflection concerns the relationship between separation anxiety and the consequent fear of the loss of the Self as opposed to shame as a consequence of a narcissistic wound linked to the Ideal Ego.

It is important to note that Self-esteem essentially represents the outcome of the quality of primary relationships. In fact, both the psycho-dynamic and philosophical fields underline the relativity of the specific weight of low self-esteem, which represents a cause and consequence of a relational failure when it is connected to separation anxiety. Self-esteem essentially represents the outcome of the quality of primary relationships. Since the chain that leads to defense, in classical psychoanalysis, also contains excessive frustration, we could find a point of contact between Freudian theory and Kohutian theory on the origin of shame. In this regard, the modulation of the presence of the other in the various theories is noteworthy. For Lacan (2007b), shame follows the gaze of the other, real or imaginary. However, for Kohut (1980), this affect would result from the absence of the object's gaze in the face of one's exhibitionism. Shame-proneness begins when a child needs to be mirrored but is not, and this lack of internalized positive mirroring experiences might sensitize the child to later shame.

The link between shame and the body is notable. In fact, this archaic affect seems to strongly involve the body, as explicitly highlighted by Sartre, and by the symptoms inherent in the body in the various pathologies. For example, it is present explicitly in eating disorders such as anorexia and obesity, and also in specific symptoms of depression and schizophrenia.

In psychosis, conscious shame can present itself in excess or in defect, determining, respectively, the negative and positive symptoms. To illustrate, there is often a pathological lack of shame, a shamelessness, that does not seem to allow an encounter with the other. This absence seems to be a consequence of the fact that the psychotic is completely identified with shame, “he becomes the shame” (Termini, 2018). As noted above, one can probably find a link between becoming ashamed and Binswanger's idea that the positioning of the center of gravity of one's existence is in line with the judgment of others, which is not fluid but permanently fixed. Regarding the lack of shame in psychosis, a question can be asked whether it is attributable to regression or, rather with a higher probability, to a defensive constellation that relegates this affect to an unconscious level, thus also preventing the emergence of the anguish signals of shame.

As psychiatrists, we have long known that psychotics, referred to in past centuries as “alienated”, are precisely those who sometimes shamelessly break cultural norms. For example, sometimes it appears as if they have no problem disrobing in public or talking to anyone about what most horrifies them. Can we perhaps use the presence or absence of shame in the course of the psychiatric patient's story in what concerns him or her as a diagnostic index to identify the psychotic structure? And can the emergence of a veil of shame in psychotic patients be read as an indication of the therapeutic result?

Another element to consider is that shame in its positive meaning seems to be connected to the human need for a relationship with the other, which plays a fundamental role in everyone's identity. Both an excess and a lack of shame can characterize the pathological shame that seems to derive from unfulfilled primary needs (in particular the mirror and twin needs) and the consequent separation anxiety. Relational renunciation, typical of depression, would seem to be a corollary of shame and of its defenses which are more or less all-encompassing: a “proto-defense” with a generalized blocking of emotions and activities or vigorous, albeit less complete, defenses of the sense of guilt or the angry turning on oneself.

The immodest presence of the body in eating disorders is intertwined in a circularity with the experience of shame that often represents both the cause and the consequence. The fluid dichotomy between this conscious and unconscious affect probably corresponds, respectively, to the eating disorders bulimia and anorexia, close entities at a crossroads with tenuous boundaries. This declination of shame as conscious or unconscious also seems to represent the corollary of avoidant and schizoid personality disorders, respectively. The intimate relationship between shame and narcissistic personality disorder perhaps most explicitly reveals the essence of this affect. The primary relational lack with the consequent experience of rejection overshadows the anguish of non-existence with a greedy and grandiose compensatory need.

According to Binswanger, pathological shame means that, at various levels, the center of gravity is placed outside itself, with a fixed judgment of the social context while its boundaries are weakened until they disappear in the transitivism and appersonation of the pre-psychotic phase and in the primary process (Ballerini and Rossi Monti, 2011). This dislocation of the center of gravity can also be seen in disorders where the reality principle is preserved. For example, the symptoms of Narcissistic Personality Disorder clearly show this dislocation. The unsatisfied need for the gaze of the other does not allow the construction of one's own identity which is based on identification with parents or their substitutes. This latter process, with its introjective movements, would constitute the backbone of trust in belonging to the human race.

## Author contributions

All authors listed have made a substantial, direct, and intellectual contribution to the work and approved it for publication.

## Conflict of interest

The authors declare that the research was conducted in the absence of any commercial or financial relationships that could be construed as a potential conflict of interest.

## Publisher's note

All claims expressed in this article are solely those of the authors and do not necessarily represent those of their affiliated organizations, or those of the publisher, the editors and the reviewers. Any product that may be evaluated in this article, or claim that may be made by its manufacturer, is not guaranteed or endorsed by the publisher.
